# QTL Mapping of Grain Zn and Fe Concentrations in Two Hexaploid Wheat RIL Populations with Ample Transgressive Segregation

**DOI:** 10.3389/fpls.2017.01800

**Published:** 2017-10-18

**Authors:** Leonardo A. Crespo-Herrera, Velu Govindan, James Stangoulis, Yuanfeng Hao, Ravi P. Singh

**Affiliations:** ^1^Global Wheat Program, Centro Internacional de Mejoramiento de Maíz y Trigo, Texcoco, Mexico; ^2^School of Biological Sciences, Flinders University, Adelaide, SA, Australia; ^3^Institute of Crop Sciences, Chinese Academy of Agricultural Sciences, Beijing, China

**Keywords:** wheat biofortification, QTL mapping, grain Zn, grain Fe, transgressive segregation

## Abstract

More than 50% of undernourished children live in Asia and more than 25% live in Africa. Coupled with an inadequate food supply, mineral deficiencies are widespread in these populations; particularly zinc (Zn) and iron (Fe) deficiencies that lead to retarded growth, adverse effects on both the immune system and an individual's cognitive abilities. Biofortification is one solution aimed at reducing the incidence of these deficiencies. To efficiently breed a biofortified wheat variety, it is important to generate knowledge of the genomic regions associated with grain Zn (GZn) and Fe (GFe) concentration. This allows for the introgression of favorable alleles into elite germplasm. In this study we evaluated two bi-parental populations of 188 recombinant inbred lines (RILs) displaying a significant range of transgressive segregation for GZn and GFe during three crop cycles in CIMMYT, Mexico. Parents of the RILs were derived from *Triticum spelta* L. and synthetic hexaploid wheat crosses. QTL analysis identified a number of significant QTL with a region denominated as *QGZn.cimmyt-7B_1P2* on chromosome 7B explaining the largest (32.7%) proportion of phenotypic variance (PVE) for GZn and leading to an average additive effect of −1.3. The QTL with the largest average additive effect for GFe (−0.161) was found on chromosome 4A (*QGFe.cimmyt-4A_P2*), with 21.14% of the PVE. The region *QGZn.cimmyt-7B_1P2* co-localized closest to the region *QGZn.cimmyt-7B_1P1* in a consensus map built from the linkage maps of both populations. Pleiotropic or tightly linked QTL were also found on chromosome 3B, however of minor effects and PVE between 4.3 and 10.9%. Further efforts are required to utilize the QTL information in marker assisted backcrossing schemes for wheat biofortification. A strategy to follow is to intercross the transgressive individuals from both populations and then utilize them as sources in biofortification breeding pipelines.

## Introduction

Malnutrition in its different forms affects more than 2 billion people across the globe, and undernutrition is the main cause of the 45% of deaths in children aged under 5 years of age (WHO, 2017). Recent figures indicate that 155 million children suffer from stunting and 52 million are wasted, of which more than 50% live in Asia and more than 25% live in Africa (WHO, 2017); areas where there is an alarmingly high incidence of malnutrition (Muthayya et al., 2013). Mineral deficiencies, particularly Zn and Fe lead to retarded growth and affect the immune system and cognitive abilities (Bryan et al., 2004; Osendarp et al., 2007; Stoecker et al., 2009; Kambe et al., 2014). In 2016, the United Nations established a decade of action to combat malnutrition and this included the promotion and provision of healthy and sustainable food systems, encompassing investments in agriculture (WHO, 2017).

The generation of biofortified, staple food crops such as wheat, is an important opportunity to contribute to the solution of the *hidden hunger* problem in low income countries (Velu et al., 2014). Agronomic practices can contribute to wheat biofortification. For instance, it is proven that foliar applications of Zn can increase this mineral concentration in the grains, but only in conditions when the soil is Zn-deficient, and there are indications that an effective method for increasing grain Zn is the combination of soil fertilization with foliar applications (Velu et al., 2014). However, augmenting mineral concentrations solely through agronomic practices implies increased production costs for farmers, which are often not able to afford.

Genetic biofortification offers a solution that is not opposite to agronomic practices, and in fact can be synergistic (Velu et al., 2014). However, wheat improvement for higher concentrations of micronutrients in the grain requires substantial efforts in resources and money, starting from the identification of source materials with high nutrient concentration in the grain, to pre-breeding and breeding for final product development. Furthermore, the identification of favorable alleles from diverse origins is fundamental for wheat biofortification (Singh and Velu, 2017), since breeding progress requires the combination of different loci in breeding pipelines. Sources with vast genetic diversity for grain Zn and Fe concentrations, besides wheat landraces, are species such as *Aegilops tauschii* (Coss.), *Triticum monoccocum* L., *Triticum dicoccum* Schrank ex Schübl., *Triticum boeticum* Boiss., and *Triticum spelta* L. (Velu et al., 2014). The exploitation of these genetic resources can be through the development and utilization of synthetic hexaploid wheats (SHWs) to introgress favorable alleles from the tetraploid species *T. dicoccum* and the diploid species *A. tauschii* in elite bread wheats (Mujeeb-Kazi, 1995). Also favorable alles can be transferred from *T. spelta* to bread wheat by direct crossing due to the hexaploid nature of *T. spelta*. Even though, landraces, SHWs and *T. spelta* can be directly crossed with bread wheat, it requires substantial efforts to apply the appropriate selection methods to only transfer the loci of interest without losing the adaptability and yield potential of elite germplasm (Velu et al., 2012, 2014).

Modern technologies such as next-generation sequencing and advanced statistical procedures can facilitate the identification and introgression of genomic regions associated with higher Zn and Fe in the grains of elite germplasm. One way to identify genomic regions associated with traits of interest is the QTL or linkage mapping procedures. When mapping populations are developed and then phenotyped in different environments or years, it is possible to conduct QTL analysis for multi-environmental trials in various ways (Da Costa E Silva et al., 2012b; Li et al., 2015). An approach that can be utilized is inclusive-composite interval mapping (ICIM) (Li et al., 2015), where first a stepwise regression is performed in each environment to identify markers that significantly explain the phenotypic variation, which are then used to adjust the phenotypic values, and then interval mapping is conducted on the adjusted phenotypic data across environments to detect QTL with significant average additive effect, and/or QTL with significant interaction with the environment. This two-step approach has proven to be effective in controlling the genetic background effect (selection of cofactors), which in consequence reduces the variance of the estimated genetic parameter and hence increases power and precision (Li et al., 2015). The ICIM approach for multi-environmental trials gives three LOD scores, the first for the QTL-by-environment interaction, the second for the average additive effect and thirdly an overall LOD score which is the sum of the first and the second. The genome wide significance threshold can be obtained through an empirical formula or through permutation tests (Li et al., 2015).

One gene that is cloned and reported to increase Zn, Fe, and protein content in wheat grains is the *Gpc-B1* locus in chromosome 6BS, initially mapped in a population of recombinant inbred lines of tetraploid wheat (Uauy et al., 2006). *Gpc-B1* gene encodes a NAC transcription factor (NAM-B1) that accelerates senescence and increases nutrient translocation from leaves to grains (Uauy et al., 2006). Additionally, various authors have identified QTL associated with grain Zn and Fe concentrations and efficiency on various chromosomes of wheat and wheat relatives, for instance 1A, 2A, 2B, 3D, 4B, 6A, 6B, and 7A (Tiwari et al., 2009; Xu et al., 2012; Srinivasa et al., 2014; Velu et al., 2016). Some of these works have mapped QTL that are either tightly linked or are pleiotropic for grain Zn and Fe concentration, and even show some association with thousand kernnel weight (Xu et al., 2012; Hao et al., 2014; Crespo-Herrera et al., 2016). These findings are relevent, since they indicate the possibility of breeding for higher concentration of micronutrients sumultaneously. Supported by previous QTL mapping results in the Global Wheat Program at CIMMYT (Hao et al., 2014; Crespo-Herrera et al., 2016), efforts are being made to develop molecular markers associated with grain Zn and Fe concentrations in the grains, and their further validation and utilization in marker assisted backcrossing schemes.

In the present study we evaluated two diverse recombinant inbred line populations for three crop seasons. With the aid of genotyping by sequencing and QTL analysis for multi-environmental trials, we identified QTL for grain zinc and iron that may be useful for wheat biofortification.

## Materials and methods

### Plant materials

Two F6 populations (Pop1 and Pop2), each of 188 recombinant inbred lines (RILs), were developed from the cross of a synthetic hexaploid wheat (SHW) and a *T. spelta* L. derived line (Table 1). The parental lines were developed at CIMMYT for their medium-high concentration of GZn and GFe and crossed with the expectation of observing transgressive segregation in the mapping populations. Parent 1 (Table 1) of both populations was distributed in the 1st Harvest Plus Yield Trial (Velu et al., 2012), an internationally and annually distributed nursery in South Asia to CIMMYT collaborators within the International Wheat Improvement Network (IWIN).

**Table 1:** Pedigree of the parental lines used to develop two F6 mapping populations.

**Parent**	**Population 1**			**Population 2**		
	**Name**	**Pedigree**	**GID**	**Name**	**Pedigree**	**GID**
1	Bubo	PICUS/3/KAUZ^*^2/BOW//KAUZ/4/KKTS/5/**T.SPELTA PI348530**/6/ 2^*^FRANCOLIN #1	6356223	Louries	BL 1724^*^2/3/**T.DICOCCON PI272533/ AE.SQUARROSA (458)**// CMH81A.1261/ VEE#10	6354292
2	Turtur	REH/HARE//2^*^BCN/3/**CROC_1/AE. SQUARROSA (213)**//PGO /4/ HUITES /5/ **T.DICOCCON PI94624/ AE.SQUARROSA (409)**//BCN /6/REH/HARE// 2^*^BCN/3/ **CROC_1/ AE.SQUARROSA (213**)// PGO/4/HUITES/7/MUTUS	6356423	Bateleur	INQALAB 91^*^2/ TUKURU// **T.SPELTA PI348599**/3/ 2^*^INQALAB 91^*^2/KUKUNA	6356553

* *In bold are the T. spelta and SHW accessions involved in the pedigree of each parental line*.

### Phenotyping and analysis of phenotypic data

Both set of RILs were screened under field conditions at CIMMYT's experimental station in Ciudad Obregon, Sonora, Mexico (27° 37′ N, 109° 93′ W) for 3 consecutive years during the 2013–2014 (Y13-14), 2014–2015 (Y14-15), and 2015–2016 (Y15-16) growing seasons. Each RIL was sown on a double-row plot of 1 m long and 0.8 m wide in a bed-planting system; a randomized complete block design with two replicates was utilized to conduct the evaluations. During the growing seasons the minimum temperature ranged from 4.83 to 15.01°C, the maximum temperatures ranged between 21.4 and 31.94°C.

Trials were irrigated five times throughout the crop cycle and fertilized at a rate of 200–50 (N-P), of which 50–50 was applied in pre-sowing, and 150–00 at tillering stage. Diseases and pests were controlled chemically, whereas weeds were controlled manually and chemically. The soil of the field trials was previously enriched with 25 kg ha^−1^ of ZnSO_4_.7H_2_O over three crop cycles. Soil analysis of the land were RILs were grown had an average Zn concentration of 1.2 ppm at soil depth of 0–30 cm, and 0.86 ppm at a soil depth of 30–60 cm. The average Fe concentration in the soil was 5.01 and 6.1 ppm, at 0–30 and 30–60 cm soil depth, respectively.

Mineral concentrations in the grains (GZn and GFe) were measured with a “bench-top,” non-destructive, energy-dispersive X-ray fluorescence spectrometry (EDXRF) instrument (model X-Supreme 8000; Oxford Instruments plc, Abingdon, UK) standardized for high-throughput screening of GZn and GFe in whole grain wheat (Paltridge et al., 2012).

All analyses of the phenotypic data were conducted in R v3.3.2 (R Development Core Team, 2013) with the lme4 package (Bates et al., 2015). Best linear unbiased predictors (BLUPs) of each RIL were obtained for single crop cycles by specifying a single year analysis model where RILs and reps were regarded as random effects. Additionally BLUPs across years of evaluation were obtained by specifying a multi-year analysis model, where RILs, RILs × year interaction and reps within year were regarded as random effects. Heritability (h^2^) was computed from the variance components. In addition, to assess the significance of the genotype-by-year interaction (GxY), we performed an analysis of variance with the RILs, years and the interaction of these as fixed effects.

### Genotyping

Populations were genotyped with the Diversity Array Technology (DArT), and DArT-Seq. The array technology reduces the DNA complexity by using a combination of restriction enzymes to obtain a representation of the whole genome; the variable fragments of DArT are hybridized to a library of the species of interest, thus showing its nature of “presence/absence” patterns (Wenzl et al., 2004). The difference between DArT and DArT-Seq is that the latter works with the next generation sequencing technologies and skips the hybridization process, thus greater amounts of marker information can be obtained (Sansaloni et al., 2011). SNP calling was made simultaneously for both populations.

### Linkage analysis and QTL mapping

A total of 9,034 markers were obtained for each population after the genotyping procedures. The markers that were not retained for the linkage analysis were those with: more than 20% missing data, minor allele frequency lower than 5% and those that were monomorphic between the parents of each population. Linkage and QTL analyses were conducted with the ICIMapping software (Li et al., 2008; Meng et al., 2015). The chromosome location of DArT markers (Akbari et al., 2006) was used as anchoring information to group the DArT-Seq markers using a LOD = 5.0 as significance threshold, in this way the markers with unknown chromosome assignment (DArT-Seq) can be grouped together with those that have chromosome information (DArT) given the indicated significance threshold. Markers were ordered with the Traveling Salesman algorithm, using a 5 cM window size for rippling the markers in linkage groups (LGs). Linkage groups with less than three markers or markers with no linkage were discarded from the analysis. The LG for Pop1 were built with 5,301 markers, of which 4,120 were DArT-Seq. The LG for Pop2 were constructed with 4,875 markers, of which 4,521 were DArT-Seq. Consensus maps derived from both populations were built for chromosomes that commonly harbored genomic regions associated with GZn and GFe. The consensus maps were built with the package LPmerge (Endelman and Plomion, 2014) in the R software R v3.3.2.

Inclusive composite interval mapping (ICIM) was used to make QTL analysis. The ICIM method applies a strategy in which a stepwise regression is firstly made, so markers with significant effect on QTL are selected, and then follows an interval mapping step, where the phenotypic values are adjusted by the selected marker variables, except for the two markers that flank the scanning position at each mapping step for background control. ICIM was performed with the multi-environmental model built in ICIMapping (Li et al., 2015). Significant LOD thresholds were taken at the 5% tail of the null distribution in a 1,000 permutations test (Da Costa E Silva et al., 2012a,b).

After the QTL analysis a search was conducted with the Basic Local Alignment Search Tool (BLAST) in the Ensemble Plants database of the bread wheat genome (http://plants.ensembl.org/index.html) with the default provided parameters. The search was conducted with the sequence of the markers flanking the QTL.

## Results

### Phenotypic evaluations

The levels of GZn in the progenitors of Pop1 ranged from 43.7–61.7 to 31.1–35.6 mg·kg^−1^ for GFe, while the range of GZn in the progenitors of Pop2 was 49.5–66.2 and 33.2–37.6 mg·kg^−1^ for GFe (Table 2).

**Table 2:** Grain Zn and Fe concentrations (mg·Kg^−1^) ± SE of the parents of two F6 mapping populations evaluated in 3 years.

**Population**	**Parent**	**GZn**				**GFe**			
		**Y13-14**	**Y14-15**	**Y15-16**	**Across Years**	**Y13-14**	**Y14-15**	**Y15-16**	**Across Years**
Pop1	Bubo	54.9 ± 2.1	43.7 ± 1.6	45.7 ± 1.6	48.1 ± 1.4	31.2 ± 0.88	31.1 ± 0.83	34.1 ± 0.92	31.7 ± 0.65
	Turtur	61.7 ± 2.1	47.2 ± 1.6	48.1 ± 1.6	52.4 ± 1.4	34.6 ± 0.88	32.8 ± 0.83	35.6 ± 0.92	35.0 ± 0.65
Pop2	Loruries	57.8 ± 2.9	49.5 ± 2.69	57.7 ± 2.5	63.4 ± 2.2	35.1 ± 0.99	33.2 ± 0.84	37.6 ± 0.98	35.5 ± 0.74
	Bateleur	66.2 ± 2.9	49.7 ± 2.69	54.4 ± 2.5	64.8 ± 2.2	34.5 ± 0.99	33.5 ± 0.84	36.0 ± 0.98	35.0 ± 0.74

**Figure 1:**
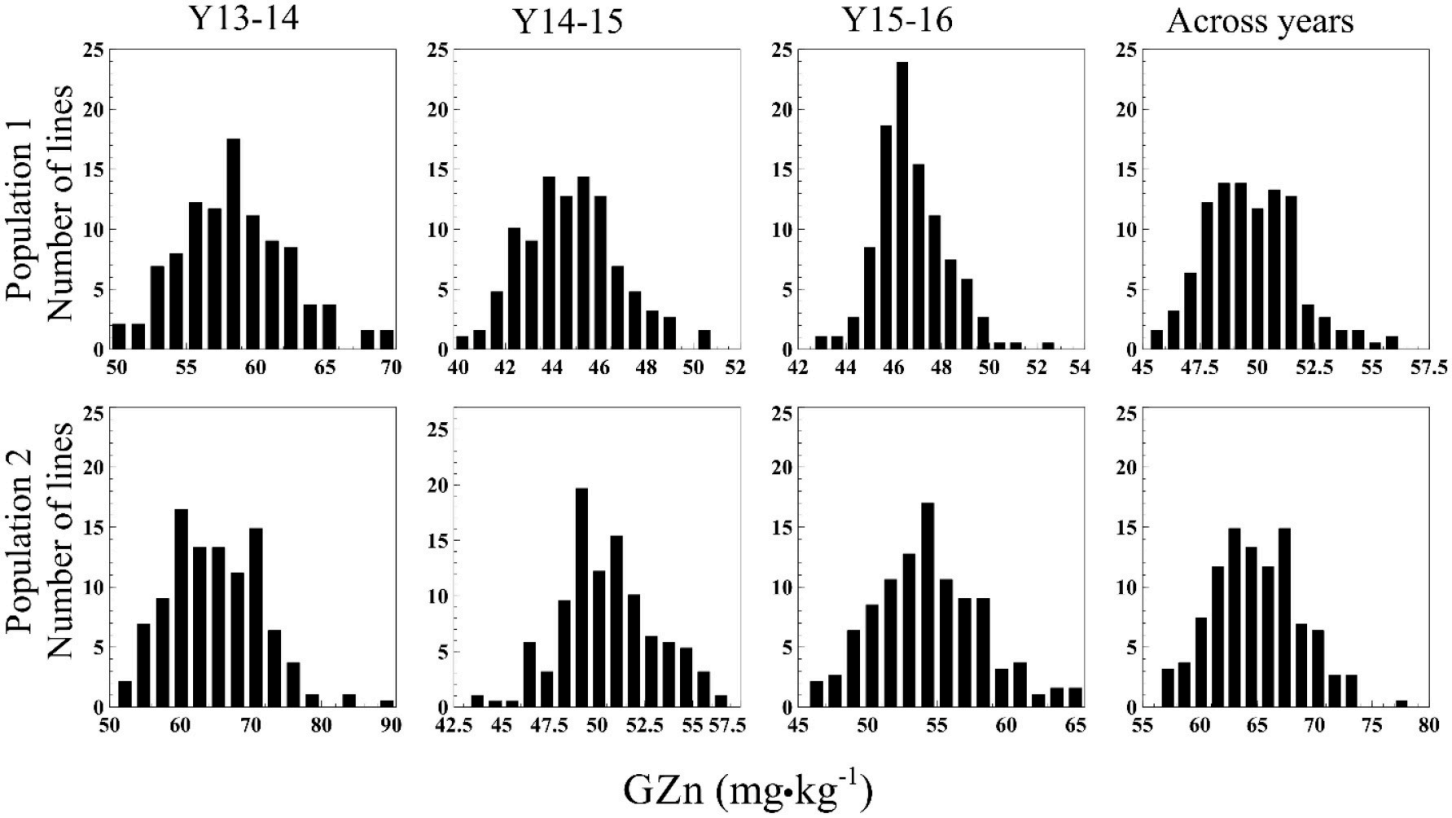
Histograms of GZn in two mapping populations of recombinant inbreed lines evaluated during 3 years, and across years.

**Figure 2:**
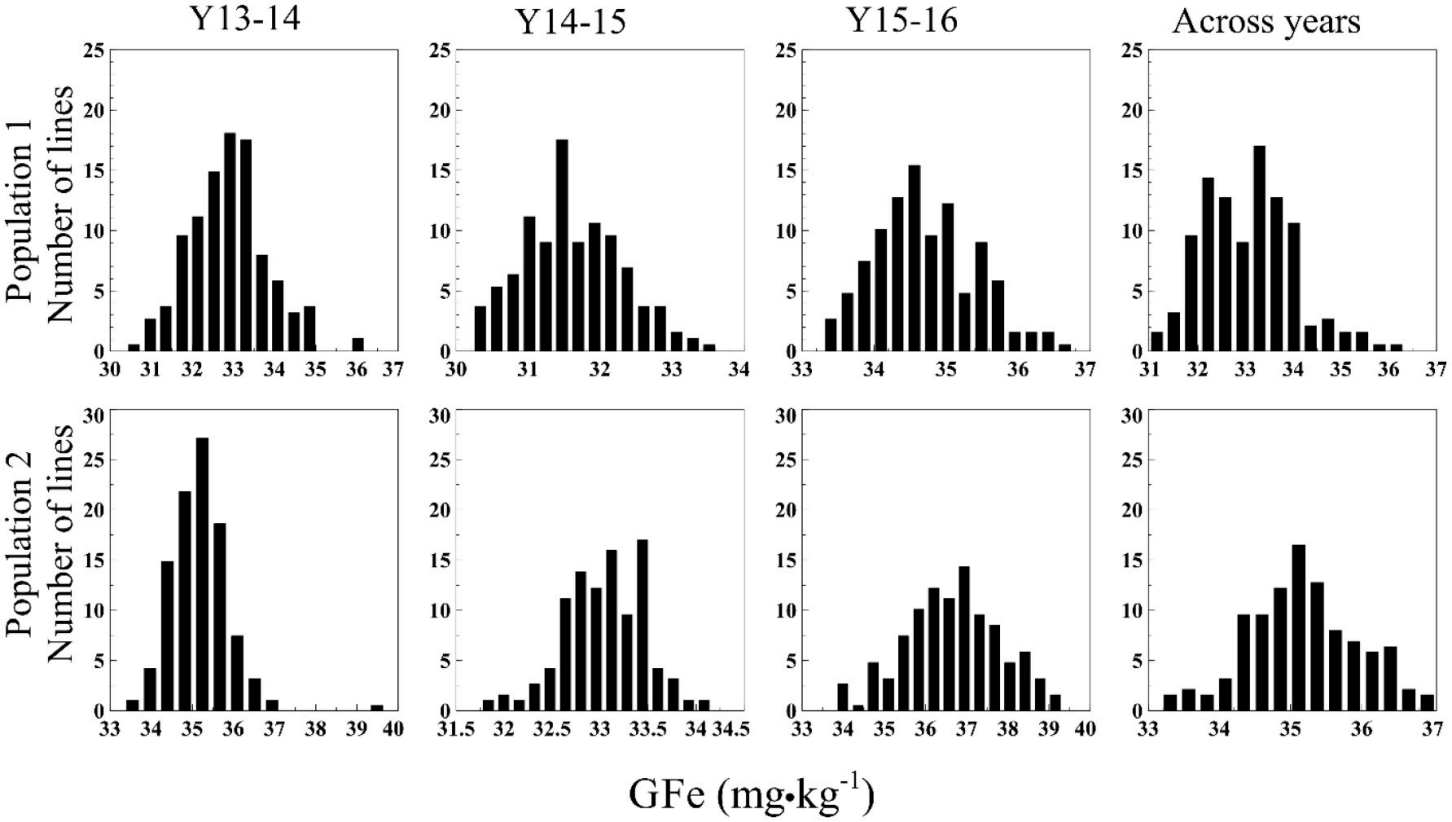
Histograms of GFe in two mapping populations of recombinant inbreed lines evaluated during 3 years, and across years.

The GZn in Pop1 ranged from 49.5–70.1 mg·kg^−1^ (Y13-14), 39.8–50.8 mg·kg^−1^ (Y14-15), 42.6–52.8 mg·kg^−1^ (Y15-16), and 45.3–56.2 mg·kg^−1^ (across years) (Figure 1). The analysis of variance (ANOVA) indicated a highly significant GxY interaction [*p* < 0.0001, *F*_(374, 555.2)_ = 1.702]. The GFe varied from 30.4–36.2 mg·kg^−1^ (Y13-14), 30.2–33.6 mg·kg^−1^ (Y14-15), 33.3–36.8 mg·kg^−1^ (Y15-16), and 31.0–36.3 mg·kg^−1^ across years (Figure 2). The ANOVA indicated a non-significant GxY interaction for GFe [*p* = 0.41, *F*_(374, 555.01)_ = 1.019]. Heritability estimates for GZn were 0.73, 0.62, 0.44, and 0.49 for Y13-14, Y14-15, Y15-16, and across years, respectively, whereas the estimates for GFe were 0.54, 0.40, and 0.36 for the respective years and 0.42 across them.

In Pop2 the GZn ranged from 50.9–90.5 mg·kg^−1^ (Y13-14), 43.3–57.0 mg·kg^−1^ (Y14-15), 45.7–65.6 mg·kg^−1^ (Y15-16), and 56.5–78.3 mg·kg^−1^across years of evaluation (Figure 1). In case of GFe for Pop2, the predicted values ranged from 33.3–39.7 mg·kg^−1^ (Y13-14), 31.8–34.2 mg·kg^−1^ (Y14-15), 33.8–39.3 mg·kg^−1^ (Y15-16), and 33.2–37.0 mg·kg^−1^ for the across-years evaluation (Figure 2). The ANOVA indicated a highly significant GxY interaction for GZn [*p* < 0.0001, *F*_(374, 563)_ = 1.78] and a non-significant GxY for GFe [*p* = 0.06, *F*_(374, 561)_ = 1.15]. Heritability estimates for GZn were 0.83, 0.46, 0.70, and 0.60 for Y13-14, Y14-15, Y15-16, and across years respectively, whereas the estimates for GFe were 0.33, 0.20, and 0.57 for each respective years and 0.29 across them.

**Figure 3:**
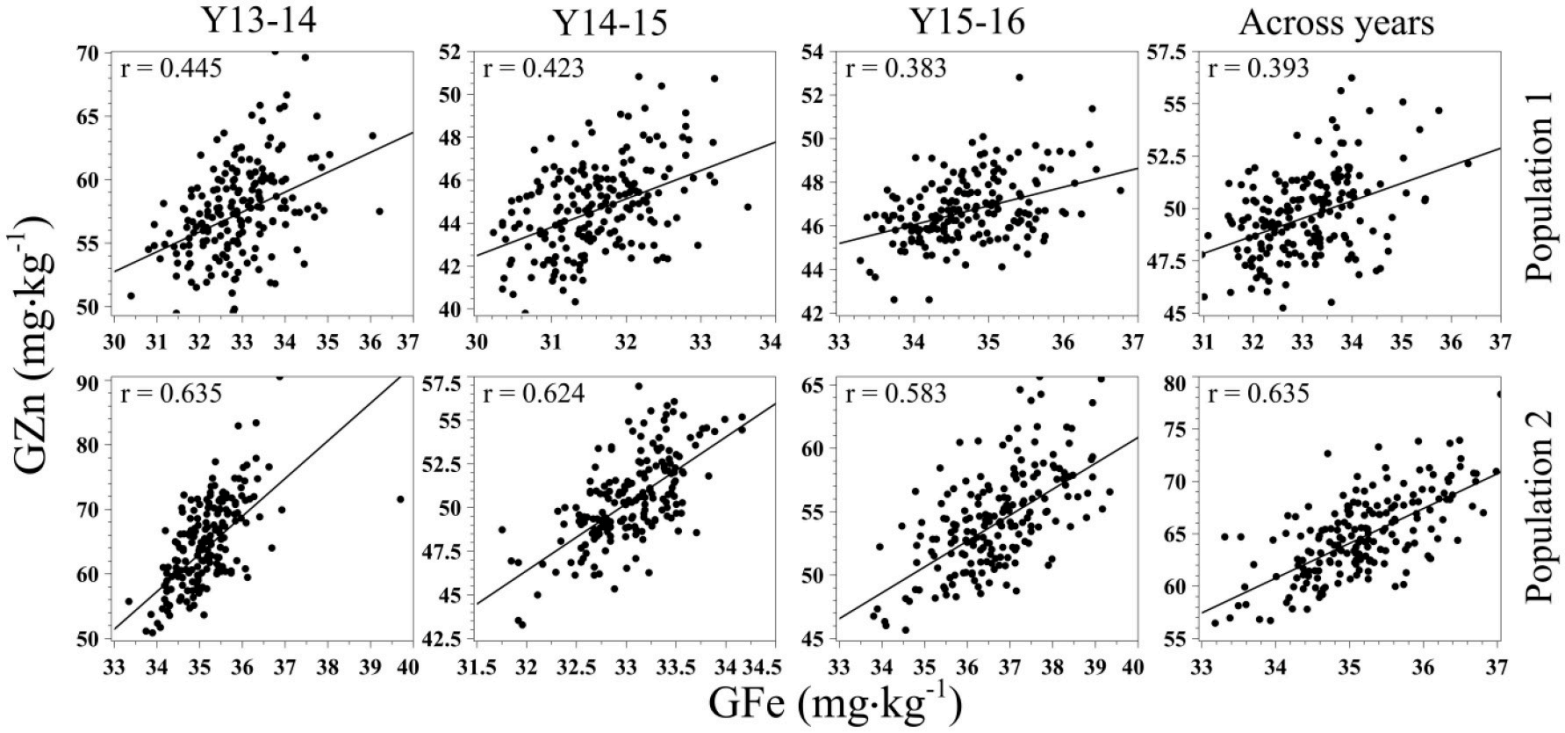
Scatter plots and correlation of GZn and GFe in two mapping populations of recombinant inbreed lines evaluated during 3 years, and across years.

Statistically significant (*p* < 0.001) correlation coefficients (*r*) were observed for each population between GZn and GFe in each year of evaluations and across years (Figure 3). Additionally, since significant GxY was identified with the ANOVA for GZn, we calculated Kendall's **τ** coefficient of concordance to determine the presence of significant rank changes, which ranged from 0.20–0.35 (*p* < 0.01) of Pop1, and 0.27–0.49 (*p* < 0.0001) for GZn of Pop2. Kendall's **τ** coefficient of concordance was not calculated for GFe as the ANOVA did not indicate significant GxY interaction.

### QTL analysis

Of the total number of markers for Pop 1 (5,301) and Pop2 (4,875), more than 50% were grouped within the B genome, whereas the D genome had the least number of markers grouped (Table 3). Linkage groups representing all wheat chromosomes were built with the genotypic data of each population. For Pop1 the number of markers in the linkage groups ranged from 24 (5D) to 635 (2B). The number of markers of the LGs for Pop2 ranged from 40 (2D) to 1,002 (1B). The map distance of the linkage groups ranged from 62.6 cM (5D) to 641 cM (3B). The average density of the linkage maps ranged from 0.5 to 1.7 markers·cM^−1^.

**Table 3:** Percentage of markers grouped by each wheat chromosome and genome in two mapping populations of recombinant inbred lines.

**Chromosome**	**1**	**2**	**3**	**4**	**5**	**6**	**7**	**Total**
**Genome**	**Population 1**							
A	5.2	3.8	5.4	4.3	1.6	7.8	7.6	35.7
B	9.3	12.0	6.8	2.5	3.8	7.9	8.6	50.9
D	1.9	1.5	3.5	0.6	0.5	2.8	2.7	13.5
	**Population 2**							
A	3.2	6.0	4.9	5.1	1.5	2.2	6.0	28.8
B	20.8	6.8	7.1	2.6	8.0	3.7	10.7	59.7
D	1.4	0.8	3.5	0.9	1.2	2.2	1.5	11.5

**Figure 4:**
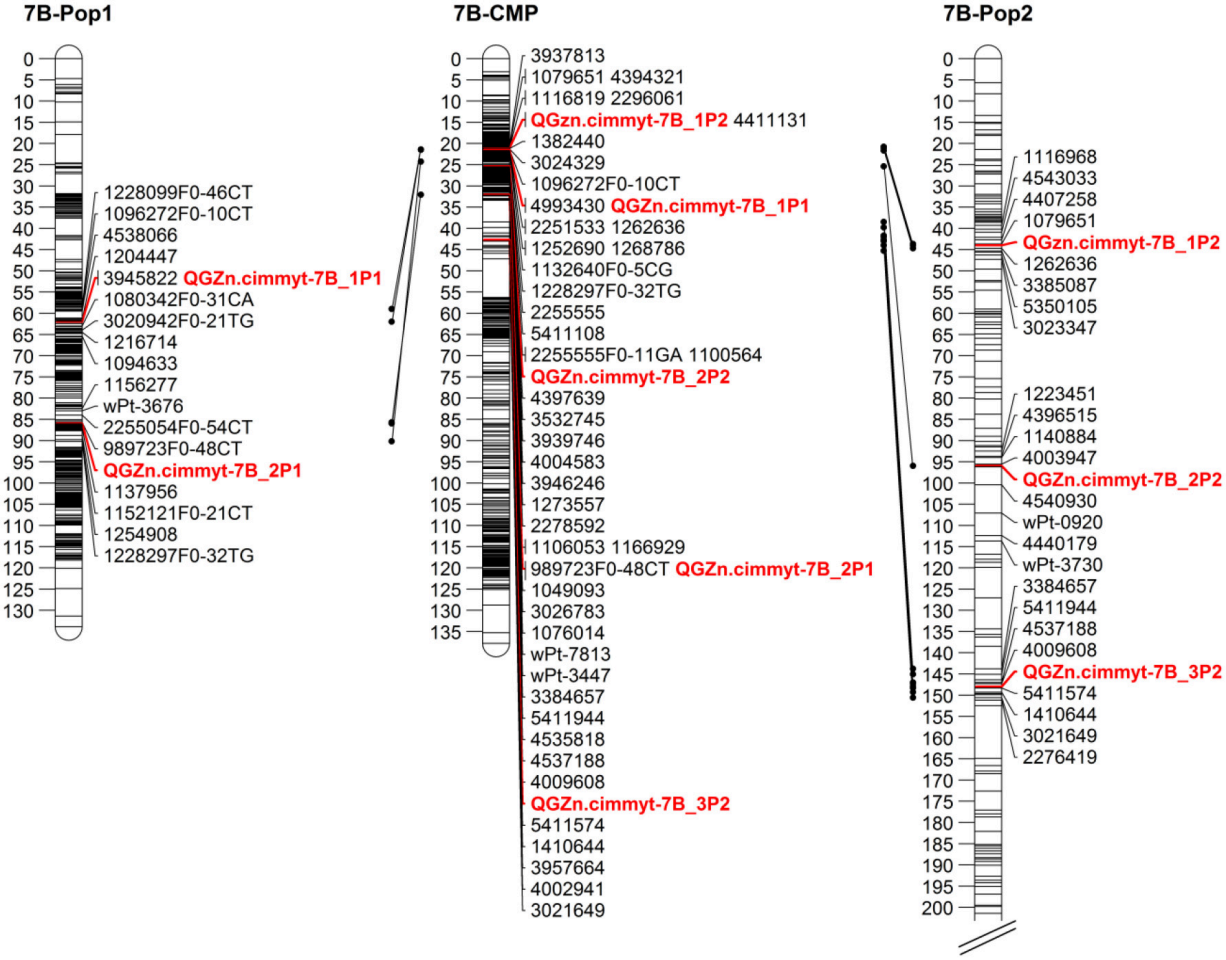
Linkage maps for chromosome 7B. 7B-Pop1: linkage map for population 1. 7B-CMP: consensus map of chromosome 7B, derived from populations 1 and 2. 7B-Pop2: linkage map for population 2. Only up to five markers flanking the QTL region are displayed.

The multi-environmental QTL analysis showed the association of various genomic regions with GZn and GFe in both populations (Tables 4, 5). The genome wide significant threshold was LOD = 4.5 and LOD = 5.0 for Pop1 and Pop2, respectively. The analysis indicated the presence of QTL for GZn on common chromosomes of both populations, namely: 1B, 6A, and 7B. In addition, one QTL for GFe in 5B was found in both populations. The consensus map built for 7B mapped the QTL for GZn from each population on near-by positions (Figure 4), indicating the presence of common regions for GZn. Consensus maps for chromosomes 1B and 6A were not possible to construct because of the reduced amount (<20%) of markers shared between linkage groups of each population.

**Table 4:** Quantitative trait loci for grain Zn (GZn) and Fe (GFe) concentrations in population 1.

**QTL**	**Position (cM)**	**Interval**	**Flanking markers**	**LOD^a^**	**LOD (Add)^b^**	**LOD (GxY)^c^**	**PVE^d^**	**PVE (GxY)^e^**	**Add^f^**	**AddxY1^g^**	**AddxY2^h^**	**AddxY3^i^**
QGZn.cimmyt-1B_P1	84	83.5–84.5	3934172;3934936	8.30	8.19	0.10	15.10	5.10	0.531	0.537	−0.256	−0.281
QGZn.cimmyt-6A_P1	109	108.5–109.5	1238392;4990410	7.91	6.49	1.42	9.71	2.22	0.457	0.326	−0.277	−0.049
QGZn.cimmyt-7B_1P1	62	61.5–62.5	3945822;1132640F0-5CG	7.12	5.41	1.71	16.75	10.40	0.424	0.766	−0.361	−0.405
QGZn.cimmyt-7B_2P1	86	85.5–86.5	989723F0-48CT; 1204955F0-26CT	5.42	1.14	4.27	2.86	1.55	0.200	−0.108	−0.195	0.303
QGFe.cimmyt-3A_P1	87	85.5–87.5	1234521;3034169F0-11AG	7.13	5.26	1.87	10.35	3.94	−0.139	−0.145	0.118	0.027
QGFe.cimmyt-4B_P1	44	43.5–44.5	1008589F0-58TC;2256263	4.56	3.77	0.79	6.69	1.99	−0.119	−0.107	0.035	0.072
QGFe.cimmyt-5B_P1	97	96.5–97.5	4989996;5410720	4.71	3.64	1.07	5.49	0.83	−0.119	0.060	−0.063	0.003

a Overall LOD score of the QTL;

b LOD score of the main additive effect;

c LOD score of the Genotype x Year interaction;

d Overall proportion of phenotypic variance explained by the QTL in percentage;

e Proportion of the phenotypic variance explained by the QTL due to Genotype x Year interaction (%);

f Main additive effect;

g,h,i *Additive x Year interaction effect for each crop cycle*.

**Table 5:** Quantitative trait loci for grain Zn (GZn) and Fe (GFe) concentrations in population 2.

**QTL**	**Position (cM)**	**Interval**	**Flanking markers**	**LOD^a^**	**LOD (Add)^b^**	**LOD (GxY)^c^**	**PVE^d^**	**PVE (GxY)^e^**	**Add^f^**	**AddxY1^g^**	**AddxY2^h^**	**AddxY3^i^**
QGZn.cimmyt-1A_P2	135	133.5–136.5	4543935;3937719	8.90	8.52	0.38	10.78	4.01	0.843	0.897	−0.617	−0.280
QGZn.cimmyt-1B_P2	227	226.5–228.5	4991478;3937490	8.58	7.81	0.77	11.25	4.93	0.814	1.004	−0.639	−0.365
QGZn.cimmyt-3B_1P2	279	277.5–284.5	3533713;1007339	5.43	4.43	1.01	4.37	1.01	0.595	0.089	−0.435	0.346
QGZn.cimmyt-3B_2P2	513	512.5–513.5	4394657;3947677	7.87	6.10	1.78	10.93	6.07	−0.717	−1.129	0.645	0.483
QGZn.cimmyt-3D_P2	193	191.5–193.5	wPt-741157;1297057	5.80	4.85	0.95	7.49	3.68	0.640	0.868	−0.601	−0.268
QGZn.cimmyt-4A_P2	124	123.5–124.5	4543988;3533871	5.17	2.55	2.62	3.82	1.83	−0.455	0.233	0.380	−0.613
QGZn.cimmyt-5B_P2	106	102.5–108.5	1078595;4538122	6.60	4.29	2.31	5.05	1.88	−0.576	−0.010	0.549	−0.540
QGZn.cimmyt-6A_P2	179	178.5–179.5	1697218;1082136	11.88	9.60	2.29	8.53	0.81	−0.907	0.104	0.296	−0.400
QGZn.cimmyt-7B_1P2	44	43.5–44.5	1079651;1262636	20.76	18.41	2.34	32.79	17.16	−1.290	−1.905	1.101	0.804
QGZn.cimmyt-7B_2P2	96	95.5–98.5	4003947;3532745	7.77	2.94	4.83	3.30	0.99	−0.493	0.363	−0.423	0.060
QGZn.cimmyt-7B_3P2	148	146.5–148.5	4009608;5411574	7.03	3.29	3.74	5.40	2.82	−0.527	0.180	0.566	−0.745
QGZn.cimmyt-7D_P2	48	41.5–53.5	wPt-733859;3033815	5.23	4.72	0.51	5.81	2.42	−0.596	−0.706	0.418	0.288
QGFe.cimmyt-2A_P2	425	420.5–428.5	4262668;1226245	6.36	3.94	2.42	14.23	9.50	0.112	−0.107	−0.118	0.225
QGFe.cimmyt-2B_P2	172	170.5–173.5	wPt-0289;1026059	4.98	3.22	1.76	5.79	1.88	0.102	0.091	−0.080	−0.011
QGFe.cimmyt-3B_1P2	278	275.5–281.5	3533713;1007339	5.10	2.89	2.21	5.81	2.31	0.097	0.106	−0.083	−0.023
QGFe.cimmyt-3B_2P2	513	511.5–513.5	4394657;3947677	6.52	2.09	4.43	7.19	4.64	−0.083	−0.158	0.071	0.087
QGFe.cimmyt-4A_P2	199	198.5–199.5	3385350;1211533	9.65	7.86	1.79	21.14	11.41	−0.161	0.140	0.106	−0.245
QGFe.cimmyt-4D_P2	1	0–2.5	2363822;3961236	6.45	3.58	2.87	14.62	10.23	−0.109	0.139	0.095	−0.234
QGFe.cimmyt-5B_P2	173	168.5–174.5	4407677;1129284	5.38	3.04	2.34	11.62	8.08	−0.097	0.098	0.109	−0.207

a Overall LOD score of the QTL;

b LOD score of the main additive effect;

c LOD score of the Genotype x Year interaction;

d Overall proportion of phenotypic variance explained by the QTL in percentage;

e Proportion of the phenotypic variance explained by the QTL due to Genotype x Year interaction (%);

f Main additive effect;

g,h,i *Additive x Year interaction effect for each crop cycle*.

The proportion of the total phenotypic variance (PVE) explained by the QTL for GZn in Pop1 ranged from 2.86–16.75%, and 5.49–10.35% for GFe (Table 4). On the other hand, the PVE of the QTL for GZn in Pop2 ranged from 3.3–32.79%, and 5.79–21.14% for GFe (Table 5). The QTL that had the highest PVE for GZn in Pop1 was *QGZn.cimmyt-7B_P1* (16.75%), and in Pop2 was *QGZn.cimmyt-7B_1P2* (32.7%). For GFe, the QTL with the highest PVE were *QFe.cimmyt-3A_P1* (10.35%) and *QGFe.cimmyt-4A_P2* (21.14%) for Pop1 and Pop2, respectively.

**Figure 5:**
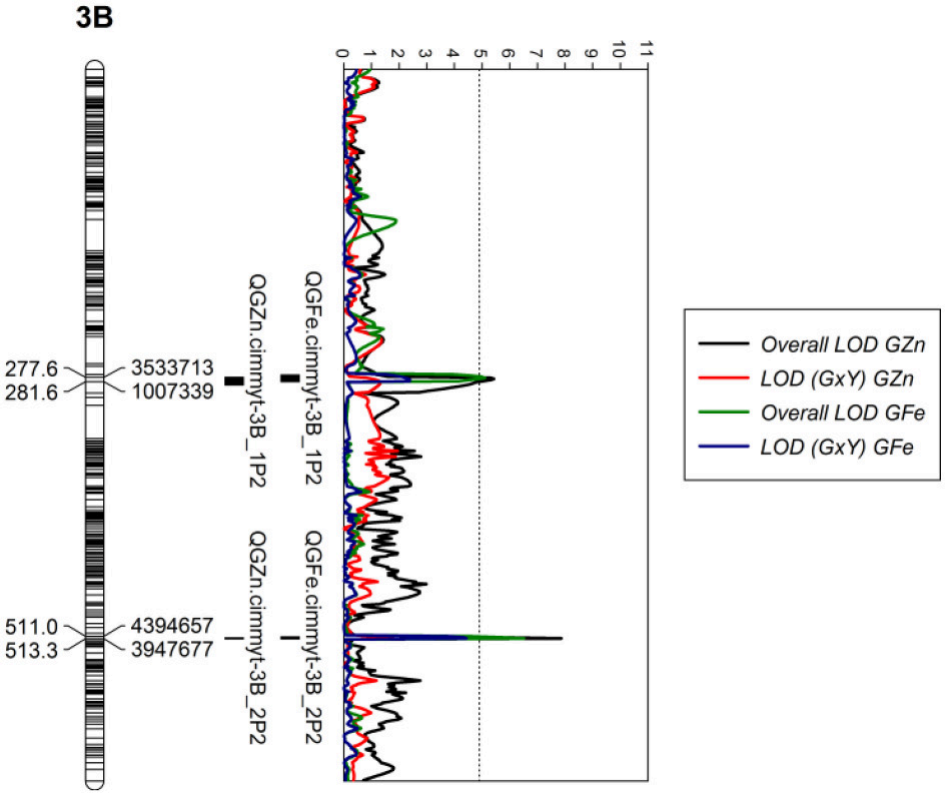
LOD profiles of chromosome 3B for GZn and GFe in population 2. The dotted line indicates the genome-wide significance threshold after a run of 1,000 permutations.

**Figure 6:**
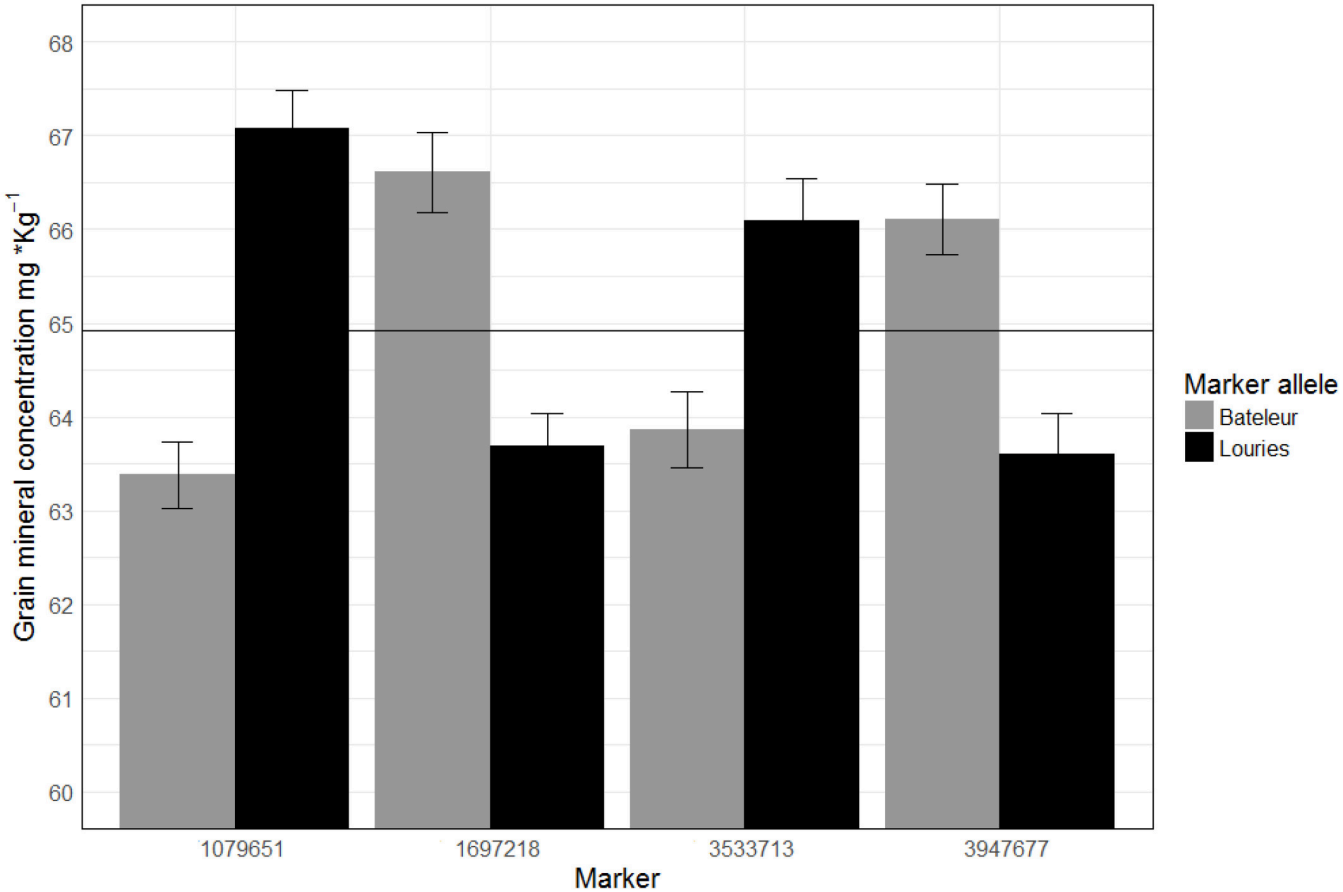
Average of GZn across three years of evaluations in RILs of population 2 classified by marker alleles linked to different QTL. The horizontal line represents the observed average (64.91 mg·Kg^−1^) of GZn in population 2 across years.

We localized two genomic regions with pleiotropic effects on chromosome 3B for GZn and GFe of Pop2 (Figure 5), both regions with minor effects (Figure 6, Table 5). The first one for GZn (*QGZn.cimmyt-3B_1P2*) and GFe (*QGFe.cimmyt-3B_1P2*), linked to marker 3533713 between the interval of 275.5–284.5 cM (Table 5). The region derived from Louries (Parent 1, Pop2) and explained 4.37 and 5.81% of the PVE for GZn and GFe, respectively (Table 5). The second region, from Bateleur (Parent 2, Pop 2), was associated to *QGZn.cimmyt-3B_2P2* and *QGFe.cimmyt-3B_2P2*, linked to marker 3947677, between the interval 511.5–513.5 cM, with a PVE of 10.93 and 7.19% of GZn and GFe, respectively.

The *QGZn.cimmyt-7B_1P2* region linked to marker 1079651 had the largest PVE for GZn (32.7%) on chromosome 7B from Louries, which was also displayed the highest additive effect of all QTL (−1.29) (Figure 6, Table 5). Whereas, for GFe the QTL with the largest PVE (21.14%) was *QGFe.cimmyt-4A_P2* on chromosome 1B (Table 5).

The QTL with the second largest average additive effect was found on chromosome 6A (*QGZn.cimmyt-6A_P2*) between the interval 178.5–179.5, linked to marker 1697218, which explained a total PVE of 8.53%. Additional QTL of interest are those found on chromosomes 1A and 1B (Table 5), which displayed 10.78 and 11.25% of PVE, respectively.

### Sequence alignment (BLAST)

The sequence of the flanking marker of each QTL were entered in the Ensambl Plants database of the bread wheat genome sequence (http://plants.ensembl.org/index.html). Of all the QTL identified, 10 were located where genes coding for uncharacterized proteins are present and/or no-gene annotation is available. The rest of the QTL appeared to be located in regions were genes coded for diverse proteins, including mainly: Leucine-rich repeat (4 QTL), P-loop containing nucleoside triphosphate hydrolase (3 QTL), Peptidase C13 and Peptidase M41 (2 QTL), Protein kinase (3 QTL), Cytochrome P450 (4 QTL). Other coded proteins found were: Winged helix DNA-binding, Glycosyltransferase, Homeobox KN domain, Multicopper oxidases, Concanavalin A-like lectin/glucanase, Xyloglucan endo-transglycosylase, Zinc finger and Zinc knuckle CX2CX4HX4C (Table 6).

**Table 6:** Coded proteins by genes present in the genomic region where QTL for GZn and GFe were found in two RIL populations.

**QTL**	**Population**	**Coded protein**
QGZn.cimmyt-1B_P1	1	Endo/exonuclease/phosphatase
QGZn.cimmyt-6A_P1	1	Leucine-rich repeat; P-loop containing nucleoside triphosphate hydrolase; Winged helix DNA-binding
QGZn.cimmyt-7B_1P1	1	Putative gypsy type transposon^*^; Peptidase_C65_otubain; Protein kinase
QGZn.cimmyt-7B_2P1	1	Glycosyltransferase;
QGFe.cimmyt-3A_P1	1	Peptidase C13
QGFe.cimmyt-4B_P1	1	Homeobox KN domain; POX domain
QGFe.cimmyt-5B_P1	1	6-phosphogluconolactonase domain; Glucosamine/galactosamine-6-phosphate isomerase; Rab-GTPase-TBC domain
QGZn.cimmyt-1A_P2	2	Protein kinase; Multicopper oxidases
QGZn.cimmyt-1B_P2	2	^*^
QGZn.cimmyt-3B_1P2	2	^*^
QGZn.cimmyt-3B_2P2	2	Cytochrome P450
QGZn.cimmyt-3D_P2	2	^*^
QGZn.cimmyt-4A_P2	2	^*^
QGZn.cimmyt-5B_P2	2	^*^
QGZn.cimmyt-6A_P2	2	^*^
QGZn.cimmyt-7B_1P2	2	Concanavalin A-like lectin/glucanase; Xyloglucan endo-transglycosylase
QGZn.cimmyt-7B_2P2	2	Leucine-rich repeat; Protein kinase; EF-Hand 1
QGZn.cimmyt-7B_3P2	2	Zinc finger;Zinc knuckle CX2CX4HX4C; Leucine-rich repeat; Protein prenyltransferase
QGZn.cimmyt-7D_P2	2	–
QGFe.cimmyt-2A_P2	2	Cytochrome P450; Peptidyl-prolyl cis-trans isomerase, FKBP-type; Protein kinase; Bulb-type lectin; S-receptor-like serine/threonine-protein kinase; WRKY domain; Leucine-rich repeat
QGFe.cimmyt-2B_P2	2	P-loop containing nucleoside triphosphate hydrolase; Cytochrome P450
QGFe.cimmyt-3B_1P2	2	–
QGFe.cimmyt-3B_2P2	2	Cytochrome P450
QGFe.cimmyt-4A_P2	2	–
QGFe.cimmyt-4D_P2	2	–
QGFe.cimmyt-5B_P2	2	P-loop containing nucleoside triphosphate hydrolase (AAA + ATPase domain); Peptidase M41; Peptidase, FtsH

* *, Uncharacterized protein or with uknown funcion; –, Not mapped to the chromosome or no function found*.

A supplementary table is provided with the sequence of the markers flanking the QTL that was used for alignment.

## Discussion

The study of mapping populations, through the implementation of QTL analysis is useful not only to identify genomic regions associated with traits of interest, but also to utilize the information of associated markers in breeding programs to efficiently incorporate particular loci in elite germplasm. In our study, through the application of QTL-by-environment interaction with composite interval mapping (Li et al., 2015) it was possible to locate several genomic regions associated with GZn and GFe in the two mapping populations that were studied over a period of three crop cycles in northwest Mexico. Our results are in agreement with other authors' findings in that GZn and GFe are traits of quantitative nature (Tiwari et al., 2009; Xu et al., 2012; Hao et al., 2014; Srinivasa et al., 2014; Crespo-Herrera et al., 2016; Krishnappa et al., 2017). These Previous QTL studies have also mapped QTL for GZn and GFe in various chromosomes of wheat and wheat related species, inlcuding 1A, 2A, 2B, 3A, 3D, 4B, 5A, 6A, 6B, 7A, 7B, with PVE ranging from 2.3% in chromosome 5A (Krishnappa et al., 2017) to 27.1% in chromosome 3B (Srinivasa et al., 2014). In our study the largest PVE (32.79%) was displayed by *QGZn.cimmyt.7B_1P2* in chromosome 7B.

A recent QTL mapping study also identified promising genomic regions on chromosomes 1A, 2A, 2B, 5A, 7A, and 7B, with PVE ranging from 2.3 to 14.4% (Krishnappa et al., 2017). Such QTL originate from the line “*Triticum dicoccon* PI94624/*Aegilops squarrosa* [409]//BCN,” a SHW parent that is also present in the pedigree of Turutur (Parent 2, Pop2). QTL in our study were also found on those chromosomes of Pop2, except in 5A. However, it is difficult to stablish similarities between the genomic regions, because our map and that produced by Krishnappa et al. (2017) are based on different and not easily comparable type of markers (DArT vs. SSR).

The genotype by environment interaction has important implications for crop performance and breeding, particularly the cross-over type interactions. For wheat biofortification, the ideal case is to obtain stable wheat genotypes that perform well without cross-over interaction when evaluated in other environments or years in a determined geographical area. The analysis of the phenotypic data in our study indicated the presence of significant GxY interaction, however when we examined the data further and calculated the Kendall's **τ** coefficient as an indicator of rank changes in the data, we observed that **τ** values are highly significant (*p* < 0.001), particularly for GZn of both populations, which indicates a change-of-magnitude rather than a cross-over type of interaction. In addition to that, the QTL analysis showed that most of the LOD scores for the additive average effect were larger than the LOD score of the interaction (Tables 4, 5), which indicates that QTL with larger LOD (Add) are more stable than those with larger LOD (GxY) (Li et al., 2015).

Transgressive segregation was observed in both populations, particularly for GZn, given that the progenitors had similar GZn levels and the source of higher mineral concentrations are putatively of different origin, i.e., one from SHW and the other two from *T. spelta*. In fact, the coefficient of parentage (COP) between parents of Pop2 is 0.02, which is equal to the probability of genes being identical by descent, and it is calculated from the pedigree information as described by Cockerham (1983) with the Browse application of the International Crop Information System (ICIS) software described by McLaren et al. (2005). The largest range of segregation was detected in Pop2, which on average doubled that of Pop1 (COP = 0.148), and also progenitors of Pop2 contained higher GZn than the progenitors of Pop1 throughout the period of evaluation (Table 2). The fact that the progenitors of Pop2 were more distantly related than those of Pop1 could be the reason why more QTL were found in Pop2 than in Pop1. One of the attributable causes of transgressive segregation is the action of loci with complementary additive effect differentially present in parental lines, which can be observed when progenitors are distantly related (Rieseberg et al., 1999). In line with this, QTL were found to be originated from both parents, indicating the complementary effect of QTL. In addition to the finding of complementary genomic regions in the two evaluated populations, it is possible to select those transgressive individuals with ideal QTL combination to utilize them in the breeding pipeline.

*QGZn.cimmyt-7B_1P2*, detected in Pop2 and located on chromosome 7B between markers 1079651 and 1262636, was the major QTL identified in this work, with 32.8% of the PVE, and the largest additive effect (−1.29) for GZn, originated from Louries (Parent 1, Pop2). Interestingly, in the consensus map of 7B, this region co-located with another QTL (*QGZn.cimmyt-7B_1P1*) detected in Pop1, which also displayed the highest PVE in this population. However, the relationship between these two regions needs to be further validated to determine if they are the same or not. One possibility to study this relationship while the wheat genome sequence is fully annotated and complete, is by firstly developing user-friendly markers from the DArT-seq sequence and then intercrossing the lines that carry this QTL to study the seggregation pattern in F2 and F3 generations.

Even though, the assembly and annotation of bread wheat remains challenging, recent advances report a 78% genome coverage (Clavijo et al., 2017), and it was possible to align the sequence of the markers flanking the QTL with the reference sequence of the wheat genome, available in the Ensambl plants database. Various sequences overlapped or were located in regions where genes code for proteins with unknown function, low confidence coding or not annotated yet. Nevertheless, the BLAST results for *QGZn.cimmyt-1A_P2, QGZn.cimmyt-7B_2P2, QGZn.cimmyt-7B_1P1, QGFe.cimmyt-2A_P2* displayed its location in a region where genes code for the Kinase like superfamily, which catalyze phosphorylation processes in which some protein structures are Zn related (Scheeff and Bourne, 2005). Additionally, in the region of *QGZn.cimmyt-1A_P2* there was a gene encoding for Multicopper oxidases, which are reported to be involved in the uptake of Zn and Fe in green algae (Herbik et al., 2002). Furthermore, for the *QGZn.cimmyt-3B_2P2, QGFe.cimmyt-2A_P2, QGFe.cimmyt-2B_P2*, and *QGFe.cimmyt-3B_2P2*, the BLAST results showed that on such region of 3B there are genes encoding for the Cytochrome P450, which is reported to be related to Zn and Fe homeostasis, and frequently expressed under high Zn conditions in Arabidopsis (van de Mortel et al., 2006).

The QTL in 1B of both populations displayed a large PVE, 15.1% in Pop1 and 11.25% in Pop2. According to the BLAST results, *QGZn.cimmyt.1B_P1* appears to overlap with a gene that codes a protein belonging to the endo/exonuclease/phosphatase domain, which function is associated with DNA binding and repair during DNA replication (Wu et al., 2015; Kim et al., 2017). The QTL in chromosome 1B from Pop2 was located in a region where genes code for proteins with unknown function. The additional QTL found in a common chromosome were *QGZn.cimmyt.6A_P1* and *QGZn.cimmyt.6A_P2* in chromosome 6A. According to the BLAST results, *QGZn.cimmyt.6A_P1* appeared to be located in a region where genes belonging to the Leucine-rich repeat, P-loop NTPase and Winged helix DNA-binding domains are present. *QGZn.cimmyt.6A_P2* overlapped with a gene coding for a protein of unknown function. However, all these findings require further studies to determine if these regions are effectively related to our QTL findings.

We found that two genomic regions on chromosome 3B that are either pleiotropic or tightly linked for GZn and GFe. Other studies have also reported similar patterns in different chromosomes such as 2B, 4B, and 5A (Xu et al., 2012; Hao et al., 2014; Crespo-Herrera et al., 2016). These pleiotropic or tightly linked regions can partly explain the positive correlation that exists between GZn and GFe. Furthermore, this finding indicate the possibility of simultaneously breed for both traits. However, an additional, non-genetic factor that can contribute to the simultaneous allocation of Zn and Fe to the grains is nitrogen uptake. For example, in durum wheat, at high enough availability of N, Zn, and Fe are more significantly translocated to the grains (Erenoglu et al., 2011; Kutman et al., 2011).

From our analysis we conclude that the regions identified on chromosomes 7B, 6A, 3B, and 1B are of particular interest for wheat biofortification. Further efforts are required to incorporate the marker information in marker assisted backcrossing schemes. With the current information, one strategy to follow is to intercross the transgressive individuals from both populations and then utilize them as sources in the breeding pipeline, under this scheme we are currently validating and introgressing the QTL found by Crespo-Herrera et al. (2016) and Hao et al. (2014).

## Author contributions

VG and RS developed the progenitors of the population. VG, RS, and YH developed the populations. VG, JS, LC, RS, and YH, phenotyped and genotyped the population. YH and LC analyzed the data. LC wrote the main part of the manuscript. All authors discussed the data, read, edited the manuscript and approved the final version.

### Conflict of interest statement

The authors declare that the research was conducted in the absence of any commercial or financial relationships that could be construed as a potential conflict of interest.

## References

[B1] Akbari M. Wenzl P. Caig V. Carling J. Xia L. Yang S.. (2006). Diversity arrays technology (DArT) for high-throughput profiling of the hexaploid wheat genome. Theor. Appl. Genet. 113, 1409–1420. 10.1007/s00122-006-0365-4 17033786

[B2] Bates D. Mächler M. Bolker B. Walker S. (2015). Fitting linear mixed-effects models using lme4. J. Stat. Softw. 67, 1–48. 10.18637/jss.v067.i01

[B3] Bryan J. Osendarp S. Hughes D. Calvaresi E. Baghurst K. van Klinken J.-W. (2004). Nutrients for cognitive development in school-aged children. Nutr. Rev. 62, 295–306. 10.1111/j.1753-4887.2004.tb00055.x 15478684

[B4] Clavijo B. J. Venturini L. Schudoma C. Accinelli G. G. Kaithakottil G. Wright J.. (2017). An improved assembly and annotation of the allohexaploid wheat genome identifies complete families of agronomic genes and provides genomic evidence for chromosomal translocations. Genome Res. 27, 885–896. 10.1101/gr.217117.116 28420692 PMC5411782

[B5] Cockerham C. C. (1983). Covariances of relatives from relf-fertilization. Crop Sci. 23:1177 10.2135/cropsci1983.0011183X002300060035x

[B6] Crespo-Herrera L. A. Velu G. Singh R. P. (2016). Quantitative trait loci mapping reveals pleiotropic effect for grain iron and zinc concentrations in wheat. Ann. Appl. Biol. 169, 27–35. 10.1111/aab.12276

[B7] Da Costa E Silva L. Wang S. Zeng Z. B. (2012a). Composite interval mapping and multiple interval mapping: procedures and guidelines for using windows QTL Cartographer, in Quantitative Trait Loci (QTL): Methods and Protocols Methods in molecular biology, ed Rifkin S. A. (New York, NY: Humana Press), 75–119. 10.1007/978-1-61779-785-9_6 22565834

[B8] Da Costa E Silva L. Wang S. Zeng Z.-B. (2012b). Multiple trait multiple interval mapping of quantitative trait loci from inbred line crosses. BMC Genet. 13:67. 10.1186/1471-2156 22852865 PMC3778868

[B9] Endelman J. B. Plomion C. (2014). LPmerge: an R package for merging genetic maps by linear programming. Bioinformatics 30, 1623–1624. 10.1093/bioinformatics/btu091 24532720

[B10] Erenoglu E. B. Kutman U. B. Ceylan Y. Yildiz B. Cakmak I. (2011). Improved nitrogen nutrition enhances root uptake, root-to-shoot translocation and remobilization of zinc (^65^Zn) in wheat. New Phytol. 189, 438–448. 10.1111/j.1469-8137.2010.03488.x 21029104

[B11] Hao Y. Velu G. Peña R. J. Singh S. Singh R. P. (2014). Genetic loci associated with high grain zinc concentration and pleiotropic effect on kernel weight in wheat (*Triticum aestivum* L.). Mol. Breed. 34, 1893–1902. 10.1007/s11032-014-0147-7

[B12] Herbik A. Bölling C. Buckhout T. J. (2002). The involvement of a multicopper oxidase in iron uptake by the green algae *Chlamydomonas reinhardtii*. Plant Physiol. 130, 2039–2048. 10.1104/pp.013060 12481087 PMC166715

[B13] Kambe T. Hashimoto A. Fujimoto S. (2014). Current understanding of ZIP and ZnT zinc transporters in human health and diseases. Cell. Mol. Life Sci. 71, 3281–3295. 10.1007/s00018-014-1617-0 24710731 PMC11113243

[B14] Kim H.-S. Nickoloff J. A. Wu Y. Williamson E. A. Sidhu G. S. Reinert B. L.. (2017). Endonuclease EEPD1 is a gatekeeper for repair of stressed replication forks. J. Biol. Chem. 292, 2795–2804. 10.1074/jbc.M116.758235 28049724 PMC5314175

[B15] Krishnappa G. Singh A. M. Chaudhary S. Ahlawat A. K. Singh S. K. Shukla R. B.. (2017). Molecular mapping of the grain iron and zinc concentration, protein content and thousand kernel weight in wheat (*Triticum aestivum* L.). PLoS ONE 12:e0174972. 10.1371/journal.pone.0174972 28384292 PMC5383102

[B16] Kutman U. B. Yildiz B. Cakmak I. (2011). Effect of nitrogen on uptake, remobilization and partitioning of zinc and iron throughout the development of durum wheat. Plant Soil 342, 149–164. 10.1007/s11104-010-0679-5

[B17] Li H. Ribaut J.-M. Li Z. Wang J. (2008). Inclusive composite interval mapping (ICIM) for digenic epistasis of quantitative traits in biparental populations. Theor. Appl. Genet. 116, 243–260. 10.1007/s00122-007-0663-5 17985112

[B18] Li S. Wang J. Zhang L. (2015). Inclusive composite interval mapping of QTL by environment interactions in biparental populations. PLoS ONE 10:e0132414. 10.1371/journal.pone.0132414 26161656 PMC4498613

[B19] McLaren C. G. Bruskiewich R. M. Portugal A. M. Cosico A. B. (2005). The international rice information system. A platform for meta-analysis of rice crop data. Plant Physiol. 139, 637–642. 10.1104/pp.105.063438 16219924 PMC1255983

[B20] Meng L. Li H. Zhang L. Wang J. (2015). QTL IciMapping: integrated software for genetic linkage map construction and quantitative trait locus mapping in biparental populations. Crop J. 3, 269–283. 10.1016/j.cj.2015.01.001

[B21] Mujeeb-Kazi A. (1995). Interspecific crosses: hybrid production and utilization, in Utilizing Wild Grass Biodiversity in Wheat Improvement: 15 Years of Wide Cross Research at CIMMYT, eds Mujeeb-Kazi A. Hettel G. P. (Mexico, D.F: CIMYMT), 14–21.

[B22] Muthayya S. Rah J. H. Sugimoto J. D. Roos F. F. Kraemer K. Black R. E. (2013). The global hidden hunger indices and maps: an advocacy tool for action. PLoS ONE 8:e67860. 10.1371/journal.pone.0067860 23776712 PMC3680387

[B23] Osendarp S. J. Baghurst K. I. Bryan J. Calvaresi E. Hughes D. Hussaini M.. (2007). Effect of a 12-mo micronutrient intervention on learning and memory in well-nourished and marginally nourished school-aged children: 2 Parallel, randomized, placebo-controlled studies in Australia and Indonesia. Am. J. Clin. Nutr. 86, 1082–1093. 17921387 10.1093/ajcn/86.4.1082

[B24] Paltridge N. G. Palmer L. J. Milham P. J. Guild G. E. Stangoulis J. C. R. (2012). Energy-dispersive X-ray fluorescence analysis of zinc and iron concentration in rice and pearl millet grain. Plant Soil 361, 251–260. 10.1007/s11104-011-1104-4

[B25] R Development Core Team (2013). R: A Language and Environment for Statistical Computing. Vienna: R Foundation Statistical Computing, 409.

[B26] Rieseberg L. H. Archer M. A. Wayne R. K. (1999). Transgressive segregation, adaptation and speciation. Heredity 83, 363–372. 10.1038/sj.hdy.6886170 10583537

[B27] Sansaloni C. Petroli C. Jaccoud D. Carlilng J. Detering F. Grattapaglia D. (2011). Diversity Arrays Technology (DArT) and next-generation sequencing combined: genome-wide, high throughput, highly informative genotyping for molecular breeding of Eucalyptus, in IUFRO Tree Biotechnology Conference 2011: From Genomes to Integration and Delivery, ed Grattpaglia D. (Arraial d'Ajuda: BioMed Central).

[B28] Scheeff E. D. Bourne P. E. (2005). Structural evolution of the protein Kinase-like superfamily. PLoS Comput. Biol. 1:e49. 10.1371/journal.pcbi.0010049 16244704 PMC1261164

[B29] Singh R. P. Velu G. (2017). Zinc-biofortified wheat: harnessing genetic diversity for improved nutritional quality. Sci. Br. Biofortification Ser. 1, 1–4.

[B30] Srinivasa J. Arun B. Mishra V. K. Singh G. P. Velu G. Babu R.. (2014). Zinc and iron concentration QTL mapped in a *Triticum spelta* × *T. aestivum* cross. Theor. Appl. Genet. 127, 1643–1651. 10.1007/s00122-014-2327-6 24865507

[B31] Stoecker B. J. Abebe Y. Hubbs-Tait L. Kennedy T. S. Gibson R. S. Arbide I.. (2009). Zinc status and cognitive function of pregnant women in Southern Ethiopia. Eur. J. Clin. Nutr. 63, 916–918. 10.1038/ejcn.2008.77 19190668 PMC4583768

[B32] Tiwari V. K. Rawat N. Chhuneja P. Neelam K. Aggarwal R. Randhawa G. S.. (2009). Mapping of quantitative trait Loci for grain iron and zinc concentration in diploid a genome wheat. J. Hered. 100, 771–776. 10.1093/jhered/esp030 19520762

[B33] Uauy C. Distelfeld A. Fahima T. Blechl A. Dubcovsky J. (2006). A NAC gene regulating senescence improves grain protein, zinc, and iron content in wheat. Science 314, 1298–1301. 10.1126/science.1133649 17124321 PMC4737439

[B34] van de Mortel J. E. Almar Villanueva L. Schat H. Kwekkeboom J. Coughlan S. Moerland P. D.. (2006). Large expression differences in genes for iron and zinc homeostasis, stress response, and lignin biosynthesis distinguish roots of *Arabidopsis thaliana* and the related metal hyperaccumulator *Thlaspi caerulescens*. Plant Physiol. 142, 1127–1147. 10.1104/pp.106.082073 16998091 PMC1630723

[B35] Velu G. Ortiz-Monasterio I. Cakmak I. Hao Y. Singh R. P. (2014). Biofortification strategies to increase grain zinc and iron concentrations in wheat. J. Cereal Sci. 59, 365–372. 10.1016/j.jcs.2013.09.001

[B36] Velu G. Singh R. P. Huerta-Espino J. Peña R. J. Arun B. Mahendru-Singh A. (2012). Performance of biofortified spring wheat genotypes in target environments for grain zinc and iron concentrations. Food Crop. Res. 137, 261–267. 10.1016/j.fcr.2012.07.018

[B37] Velu G. Tutus Y. Gomez-Becerra H. F. Hao Y. Demir L. Kara R. (2016). QTL mapping for grain zinc and iron concentrations and zinc efficiency in a tetraploid and hexaploid wheat mapping populations. Plant Soil 411, 81–99. 10.1007/s11104-016-3025-8

[B38] Wenzl P. Carling J. Kudrna D. Jaccoud D. Huttner E. Kleinhofs A.. (2004). Diversity Arrays Technology (DArT) for whole-genome profiling of barley. Proc. Natl. Acad. Sci. U.S.A. 101, 9915–9920. 10.1073/pnas.0401076101 15192146 PMC470773

[B39] WHO (2017). The Double Burden of Malnutrition: Policy Brief. Available online at: http://www.who.int/nutrition/publications/doubleburdenmalnutrition-policybrief/en/.

[B40] Wu Y. Lee S.-H. Williamson E. A. Reinert B. L. Cho J. H. Xia F.. (2015). EEPD1 rescues stressed replication forks and maintains genome stability by promoting end resection and homologous recombination repair. PLoS Genet. 11:e1005675. 10.1371/journal.pgen.1005675 26684013 PMC4684289

[B41] Xu Y. An D. Liu D. Zhang A. Xu H. Li B. (2012). Molecular mapping of QTLs for grain zinc, iron and protein concentration of wheat across two environments. Food Crop. Res. 138, 57–62. 10.1016/j.fcr.2012.09.017

